# Tenofovir disoproxil fumarate versus entecavir on the prognosis of hepatitis B virus-related hepatocellular carcinoma after surgical resection: a systematic review and meta-analysis

**DOI:** 10.3389/fonc.2025.1462794

**Published:** 2025-05-27

**Authors:** Yong Wang, Jia-yu Wu, Qian Xiang, Tian-fen Liao, Xiao-yan Jiang, Jing Chen

**Affiliations:** ^1^ Department of Gastrointestinal Surgery, West China Hospital, Sichuan University, Chengdu, China; ^2^ Department of Healthcare-Associated Infection Control Center, Sichuan Provincial People’s Hospital, University of Electronic Science and Technology of China, Chengdu, China

**Keywords:** tenofovir, entecavir, hepatitis B, hepatocellular carcinoma, resection

## Abstract

**Background and aim:**

Entecavir (ETV) and tenofovir disoproxil fumarate (TDF) are first-line antiviral treatment methods for chronic hepatitis B virus (HBV) infection. However, the different effects of TDF versus ETV on the prognosis of HBV-related hepatocellular carcinoma (HCC) after surgical resection remain controversial. We conducted this meta-analysis to assess the differences of TDF versus ETV in recurrence and survival for HBV-related HCC after liver resection.

**Methods:**

We searched MEDLINE, EMBASE, PubMed and Web of Science for the related studies published before January 2025. Meta-analysis was performed by use of a random-effects model.

**Results:**

A total of 15 studies were included in this meta-analysis. The pooled results showed that TDF was associated with better recurrence-free survival (RFS) (HR= 0.79, 95% CI 0.70-0.88) and lower risk of recurrence (HR=0.73, 95% CI 0.62-0.86) than ETV in HBV-related HCC patients after surgical resection. Further analysis indicated that TDF reduced the risk of late recurrence (HR= 0.70, 95% CI 0.55-0.88) rather than early recurrence (HR= 1.00, 95% CI 0.85-1.17) compared with ETV. Also, the pooled results revealed that TDF was associated with better overall survival (OS) (HR= 0.55, 95% CI 0.41-0.74) and lower risk of overall mortality (HR= 0.55, 95% CI 0.41-0.74) than ETV.

**Conclusion:**

This meta-analysis provided evidence that TDF has better benefits in improving survival and reducing late recurrence than ETV in HBV-related HCC patients after surgical resection.

## Introduction

Hepatocellular carcinoma (HCC) is one of the most common malignant tumors in the world and an important cause of cancer-related deaths (1–3). Among the risk factors for HCC, hepatitis B virus (HBV) infection is not only the main cause of HCC, but also an important prognostic indicator for lower survival rate and recurrence (4, 5). More and more evidence suggests that antiviral therapy can reduce the risk of HCC in patients with chronic HBV infection (CHB) (6).

Curative surgical resection and liver transplantation are still regarded as the most effective treatment methods for HCC at present (7–9). However, the tumor recurrence rate approximates 70% within 5 years after resection, and recurrence is most common in the first two years (10, 11). High HBV viral load is a significant independent risk factor for HCC recurrence (12–14). Previous studies showed that antiviral therapy could inhibit viral reactivation and reduce the risk of postoperative recurrence of HBV-related HCC (15, 16).

In clinical practice guidelines, entecavir (ETV) and tenofovir disoproxil fumarate (TDF) are equally recommended as first-line nucleos(t)ide analouges (NAs) for CHB, due to their high antiviral efficacy and low drug resistance rates (17, 18). However, studies comparing the effects of TDF and ETV on postoperative HCC recurrence have produced conflicting results. Some studies suggest that TDF treatment may be associated with a lower risk of recurrence than ETV (19, 20), while others report no significant differences between the two treatments in terms of recurrence and survival rates (21–23). Antiviral therapy has been shown to reduce HCC recurrence after hepatectomy for HBV-related HCC, but the effect of TDF and ETV on the prognosis of HBV-related HCC patients after resection remains controversial. Therefore, we conducted a meta-analysis to summarize the existing evidence on this topic, for providing a more reliable conclusion to guide clinical decision-making.

## Methods

### Search strategy

We systematically searched MEDLINE, EMBASE, PubMed and Web of Science databases for relevant studies up to January 2025. The following keywords were combined to search the literature: (hepatocellular carcinoma or HCC) and (surgery or resection) and (tenofovir or TDF) and (entecavir or ETV). We also manually scanned the reference lists of each paper to determine additional studies. Two authors reviewed the studies independently, and any divergences were resolved by discussion. This review was not registered.

### Study selection

Two independent reviewers evaluated the titles and abstracts of the retrieved search records, and then conduct a full-text screening of potential eligible citations. The third reviewer resolved any disagreements. The inclusion criteria were as follows, based on the PICOS framework: Population (P): Adult patients (>18 years old) diagnosed with HBV-related HCC who underwent curative surgical resection. Only studies involving patients with chronic HBV infection and primary liver cancer were considered. Intervention (I): All patients in the included studies received antiviral treatment with either entecavir (ETV) or tenofovir disoproxil fumarate (TDF) due to chronic HBV infection (CHB). Comparison (C): The studies compared the prognosis between the TDF group and the ETV group, evaluating the effects of these two antiviral treatments on postoperative outcomes. Outcome (O): The outcomes were postoperative recurrence rates and overall survival rates following surgical resection. Study Design (S): Only studies with a design of randomized controlled trials (RCTs), cohort studies, or case-control studies were included, as these study types provided the most reliable data on the efficacy of TDF versus ETV in the postoperative setting. Studies were excluded if they met one or more of the following criteria: (1) non- English published studies; (2) surgical treatments or other forms of anti-tumor treatment were performed before the curative resection, such as local ablation therapy and transarterial chemoembolization (TACE); (3) without sufficient data for analysis.

### Data extraction and quality assessment

Data were extracted independently and cross-checked by three investigators (JC, YW and QX). The article would be discussed again in case of discrepancies. The following data were extracted: first author’s name, year of publication, the country where the study was conducted, study design, sample size, number of individuals using ETV or TDF, tumor stage, and the definition of outcome.

We assessed the methodological quality of all included studies using the Newcastle Ottawa scale (NOS) for the quality assessment of cohort studies (24) and the Cochrane Handbook for randomized controlled trials (RCT) (25). The total score of each article was the sum of all items evaluated as positive. For cohort studies, NOS scores of 1−3, 4−6, and 7−9 were considered low, moderate and high−quality, respectively.

### Definitions

The primary outcomes were recurrence-free survival (RFS) and overall survival (OS) after liver resection, and the secondary outcomes were early recurrence (<2 years), and late recurrence (>=2 years) after hepatectomy. OS was defined as the duration from the date of surgical resection to the date of death from any cause, regardless of the underlying cause of death. RFS was defined as the time from the date of surgical resection to the date of the first documented recurrence of hepatocellular carcinoma (HCC) or the last follow-up in the absence of recurrence. Early recurrence was defined as the recurrence of HCC occurring within 2 years following curative surgical resection. This threshold is based on commonly accepted clinical practice, distinguishing early recurrence from late recurrence based on the time frame post-resection. Late recurrence was defined as the recurrence of HCC occurring more than 2 years after curative surgical resection.

### Statistical analysis

The results were expressed as hazard ratio (HR) and 95% confidence interval (CI). Cochran’s Q test and I^2^ index were used to evaluate the statistical heterogeneity among studies (23). P < 0.1 for Q statistic and I^2^ > 50% were considered as statistically significant heterogeneity. Once there was no significant heterogeneity, a fixed-effects model was used. Otherwise, a random-effects model was selected. Funnel plots and Egger weighted regression method were used to evaluate the publication bias, and P-value less than 0.1 was considered as a statistically significant publication bias. All statistical analyses were performed with Statistical Software-STATA, version 12.0.

## Results

### Search results

**Figure 1:**
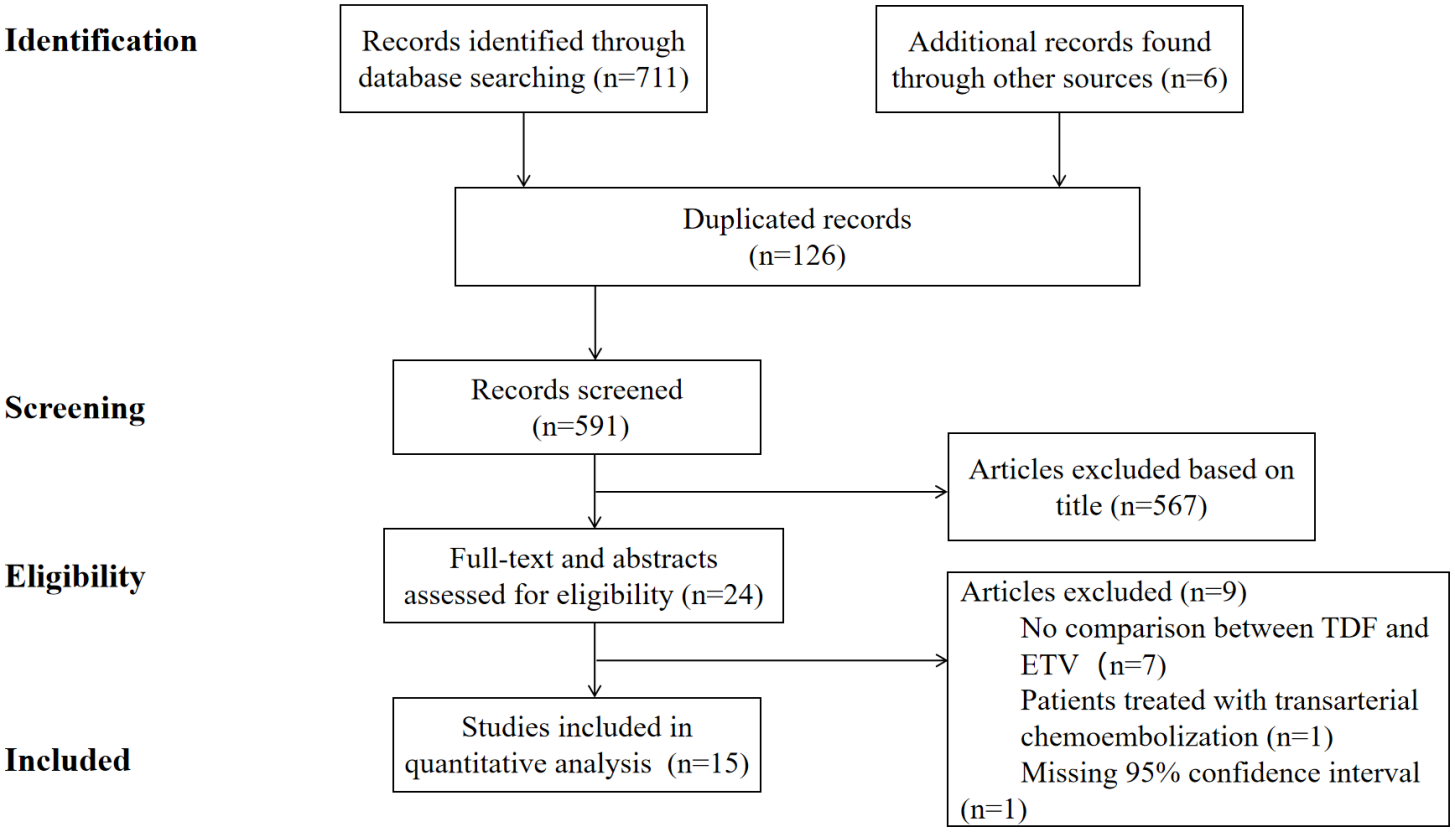
Screening and selection process of studies.

We identified 591 non-duplicate articles in the search, and 24 articles were reviewed for full text after screening the titles and abstracts of all articles. Finally, a total of 15 articles were considered eligible and were included for further assessment. The literature search process was shown in **Figure 1**.

### Study characteristics

The fifteen included studies were published between 2018 and 2025 (19–21, 23, 26–36). The detailed characteristics of the included studies were summarized in **Table 1**. All included studies were from Asia. Of the fifteen studies, one of them was randomized controlled trial (32), other fourteen were cohort studies. Among the included studies, 4 were multi-center studies (21, 26, 27, 33), and the remaining 11 were single-center studies (19, 20, 23, 28–32, 34–36). Patients of these included studies underwent surgical resection for HCC. Lee et al. (21) and Chang et al. (35) included patients who underwent liver resection or radiofrequency ablation, but we only extracted the data of patients who underwent liver resection for analysis. The sample size of TDF group and ETV group ranged from 27 to 1519 and 74 to 3462. The median follow-up period was from 28 months to 53.4 months.

**Table 1:** Characteristics of included studies.

Author, year	Study period	Study design	Setting	Sample size (TDF/ETV)	BCLC stage	Median follow-up	Outcome
**Qi et al., 2021** (26)	2014-2019	Cohort	Multi-center	144/288	0/A/B/C	47.0 months	RFS; OS
**Shen et al., 2021** (27)	2014-2019	Cohort	Multi-center	62/533	–	28.5 months	RFS
**Wu et al., 2021** (28)	2007-2018	Cohort	Single-center	42/280	0/A/B/C	48.5 months	RFS; OS
**Zhang et al., 2018** (29)	2013-2014	Cohort	Single-center	107/126	–	28.0 months	DFS
**Tsai et al., 2021** (30)	2010-2019	Cohort	Single-center	84/347	0/A	53.4 months	RFS; OS
**Wang et al., 2022** (31)	2014-2019	Cohort	Single-center	349/824	0/A/B	ETV: 42.0 months; TDF: 29.6 months	RFS; OS
**Linye et al., 2023** (32)	2017-2019	RCT	Single-center	74/74	0/A	46.61months	RFS; OS
**Choi et al., 2021** (19)	2010-2018	Cohort	Single-center	882/813	0/A	ETV: 4.4 years; TDF: 2.6 years	RFS; OS
**Lee et al., 2021** (21)	2013-2017	Cohort	Multi-center	321/405	–	ETV: 47.4 months; TDF: 44.5months	RFS; OS
**Yun et al., 2022** (33)	2011-2017	Cohort	Multi-center	1519/2040	–	3 years	RFS; OS
**Li et al., 2023** (34)	2015-2018	Cohort	Single-center	989/3462	0/A/B	51.0 months	RFS; OS
**Kao et al., 2023** (23)	2011-2016	Cohort	Single-center	432/1365	0/A/B	3.8 years	RFS; OS
**Chang et al., 2024** (35)	2011-2020	Cohort	Single-center	27/185	0/A/B	29.0 months	RFS; OS
**Chung et al., 2025** (36)	2008-2018	Cohort	Single-center	1079/1191	–	3.0 years	RFS; OS
**Kong et al., 2024** (20)	2018-2022	Cohort	Single-center	107/118	0/A/B/C	49.27 months	RFS; OS

TDF, tenofovir disoproxil fumarate; ETV, Entecavir; BCLC, Barcelona Clinic Liver Cancer; RFS, recurrence-free survival; OS, overall survival; RCT, catheter–related bloodstream infections.

### Study quality

The randomized controlled trial (32) had a low risk of bias for all items (**Supplementary Table 1**). Among the cohort studies, twelve studies had high quality (19–21, 23, 27, 29–31, 33–36), while another two studies had moderate quality (26, 28) (**Supplementary Table 2**).

### TDF versus ETV on HCC recurrence after surgical resection

**Figure 2:**
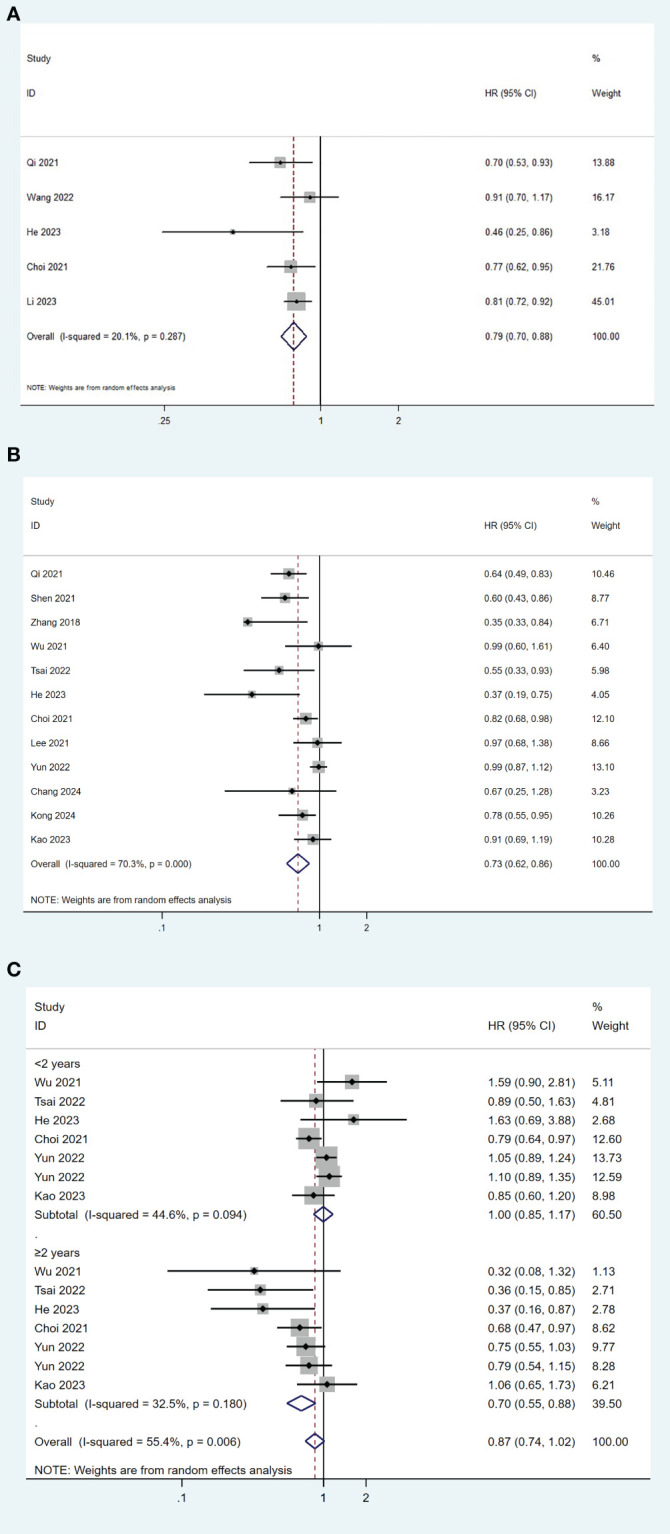
Forest plot for comparison of TDF and ETV on RFS/DFS **(A)**, risk of recurrence **(B)** and risk of early and late recurrence **(C)** in HBV-related HCC patients after surgical resection. TDF, tenofovir; ETV, entecavir; RFS, recurrence-free survival; DFS, disease-free survival; HBV, hepatitis B virus; HCC, hepatocellular carcinoma; HR, hazard ratio.

We performed the meta-analyses on all the fifteen studies for HCC recurrence. Fourteen studies (19–21, 23, 26–28, 30–36) provided HR and 95% CI for recurrence-free survival (RFS), while one study (29) provided HR and 95% CI for disease-free survival (DFS). Five studies (19, 26, 31, 32, 34) compared the differences in RFS/DFS between the TDF group and the ETV group using Kaplan–Meier analysis. The overall meta-analysis showed that TDF was associated with better RFS/DFS than ETV in HBV-related HCC patients after surgical resection (HR= 0.79, 95% CI 0.70-0.88; I^2^ = 20.1%), with a low heterogeneity among these studies (**Figure 2A**). Using the Egger weighted regression method, there was no publication bias found in this analysis (P = 0.238). Eleven studies (19, 20, 24–27, 29, 30) used the multivariable Cox regression model for further comparison, while one study (37) did not conduct further multivariate analysis due to the lack of significant results from univariate analysis. TDF was still associated with a lower risk of recurrence compared with ETV (HR= 0.73, 95% CI 0.62-0.86; I^2^ = 70.3%), with significant heterogeneity among these studies (**Figure 2B**). No publication bias was found in the analysis (P=0.800).

Further analyses were conducted by the time of recurrence. HCC recurrence after curative resection was divided into early recurrence and late recurrence based on the 2-year boundary. Six studies reported data on early and late recurrence (19, 23, 28, 30, 32, 33). The pooled results indicated that TDF treatment was significantly associated with a lower risk of late recurrence compared to ETV (HR= 0.70, 95% CI 0.55-0.88; I^2^ = 32.5%), but not with early recurrence (HR= 1.00, 95% CI 0.85-1.17; I^2^ = 44.6%) (**Figure 2C**). No publication bias was found in the analysis (P=0.173).

### TDF versus ETV on overall survival after surgical resection

**Figure 3:**
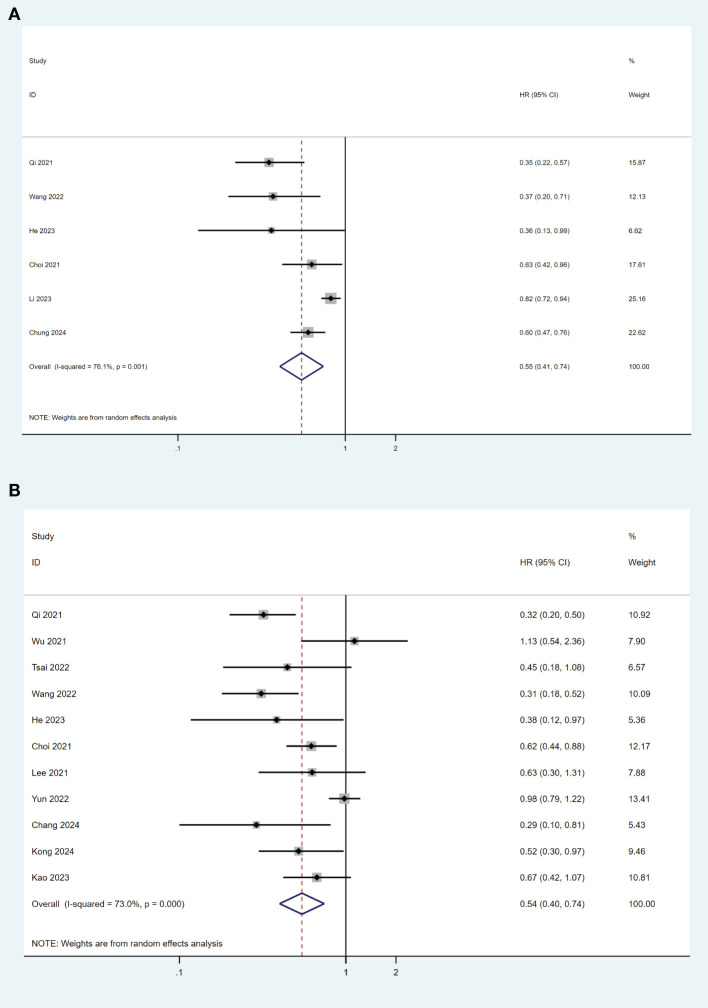
Forest plot for comparison of TDF and ETV on OS **(A)** and risk of mortality **(B)** in HBV-related HCC patients after surgical resection. TDF, tenofovir; ETV, entecavir; OS, overall survival; HBV, hepatitis B virus; HCC, hepatocellular carcinoma; HR, hazard ratio.

Overall, thirteen studies reported overall survival (OS) data (19–21, 23, 26, 28, 30–36). Among these studies, six (19, 26, 31, 32, 34) used survival analysis for comparison. The pooled results indicated that TDF was associated with better OS than ETV after surgical resection in HBV-related HCC patients (HR= 0.55, 95% CI 0.41-0.74; I^2^ = 76.1%) (**Figure 3A**). There was no publication bias found in this analysis (P = 0.213). Ten studies (19–21, 23, 26, 30–33, 35) used multivariable Cox regression model for analysis, while one study (28) only used univariate analysis. TDF was still associated with a lower risk of overall mortality compared with ETV (HR= 0.73, 95% CI 0.62-0.86; I^2^ = 70.3%), although there was significant heterogeneity among the studies (**Figure 3B**). No publication bias was found in the analysis (P=0.151).

## Discussion

Chronic hepatitis B virus infection is an important risk factor for the development of HCC, and recurrence is the most common cause of death for HCC patients after surgical resection (18, 38). Antiviral therapy has been recommended to reduce the risk of postoperative recurrence and prolong overall survival in HBV-related HCC (16, 39). Currently, TDF and ETV have been demonstrated to be effective in HBV inhibition and well tolerated (40). However, whether TDF and ETV have different clinical benefits in terms of RFS and OS in HBV-related HCC patients after resection remains controversial (41).

Our meta-analysis demonstrated that TDF was associated with better RFS and OS compared with ETV in patients undergoing curative liver resection for HBV-related HCC. The potential mechanism is currently unclear. One possible explanation for the difference in HCC recurrence or survival is that TDF and ETV have different antiviral effects. Compared to ETV, TDF treatment may be associated with higher early virological response rates and higher hepatitis B surface antigen reduction levels (42). Moreover, it has been reported that the drug resistance rate of TDF is lower than that of ETV (18). Another explanation may be that patients treated with TDF had serum interferon-λ3 (IFN-λ3) increased, while patients treated with ETV did not (43). IFN-λ showed effective anti-tumor activity in a cancer mouse model, and this anti-tumor activity might lead to difference in the risk of HCC recurrence (44–46). In addition, another study reported that TDF had additional anti-tumor effects by inhibiting the intestinal lipopolysaccharide-mediated IL-10 and inducing IL-12p70 and tumor necrosis factor (TNF)-α (47). Finally, in clinical practice in the real world, due to concerns about renal dysfunction and bone density reduction, TDF may be avoided in older adult and renal dysfunction patients with poor prognosis, leading to selection bias. If patients suffer from chronic kidney disease (CKD) or osteoporosis, according to current guidelines, priority should be given to ETV or tenofovir fumarate alafenamide (TAF). Thus, clinicians should weigh survival benefits against these risks, especially in vulnerable populations.

Due to early diagnosis and new treatment methods, the prognosis of HCC patients has gradually improved, but the long-term survival rate remains low due to the high recurrence rate of HCC after liver resection (48). Approximately 70% of patients experience recurrence within 5 years after liver resection (11). Recurrence can be divided into early recurrence within 2 years of liver resection and late recurrence after that period. The current meta-analysis indicated that TDF reduced the risk of late recurrence rather than early recurrence. Early recurrence is usually associated with factors related to primary tumor, while late recurrence may stem from *de novo* recurrence caused by the underlying liver background of hepatitis, including viral load, inflammatory activity, and degree of fibrosis (11, 49). Therefore, antiviral therapy can reduce the risk of late recurrence by inhibiting virus replication and inflammation in the liver microenvironment. TDF reduces the risk of late recurrence, which may be related to better inhibition of inflammation and viremia than ETV.

It is reported that TDF, rather than ETV, could aggravate the incidence rate of osteoporosis (50). Currently, Tenofovir fumarate alafenamide (TAF) is also recommended as a first-line drug for antiviral treatment of CHB (17). TAF is a prodrug of tenofovir and has greater stability in plasma than TDF, which promotes the increase of tenofovir concentration in hepatocytes (51). In addition, TAF can obtain similar antiviral effects at lower doses, and also reduce the incidence of side effects, including renal dysfunction and bone mineral density reduction (52). Due to the official launch of TAF after 2018 and limited research on its long-term use, it is currently unknown whether TAF is a better option for HCC patients after surgical resection. More researches are needed for exploration in the future.

In our study, heterogeneity was observed through the results of the inconsistency test (I^2^). Our analysis included a wide and diverse range of patients, varying in disease severity and duration of antiviral treatment. Three of the included studies were conducted in patients at early Barcelona Clinic Liver Cancer (BCLC) stage, two studies included patients mixed with early, intermediate, and advanced BCLC stages, and the remaining studies did not mention the HCC stage. We attempted to identify the source of heterogeneity by conducting subgroup analysis in patients with early and later BCLC stage, but the analysis was unable to be conducted due to insufficient data. In addition, populations varied in NA exposure. Some studies included NAs naive patients, some NAs experienced patients, and several did not specify treatment history. Similarly, we did not perform subgroup analysis due to the lack of specific and sufficient data. Also, follow-up periods ranged widely, potentially affecting survival outcome ascertainment.

Several limitations of our study should be taken into consideration. Firstly, most of the included studies are retrospective observational studies, and only one is RCT. So, the results should be interpreted with caution although most studies used matched cohorts for comparison. Secondly, all populations included in our study came from Asia, a region with a high prevalence of HBV-related HCC. This raises concerns about the generalizability of our findings to populations in other regions, such as Europe or North America, where the epidemiology of HBV and HCC may differ. Differences in healthcare systems and patient characteristics between regions may influence the treatment outcomes. Therefore, it remains unclear whether the results of this study can be extended to the global HBV-related HCC population. Thirdly, heterogeneity existed in the meta-analysis. The inconsistent reporting of tumor staging, terms of disease severity and duration of NA treatment might be responsible for the heterogeneity. Finally, among the included studies, some had relatively short follow-up durations. It is well known that ETV was first approved in 2005 in China, while TDF was introduced later, leading to longer follow-up in ETV groups. This temporal imbalance may bias recurrence and survival comparisons. Additionally, there was a lack of consistency in the follow-up durations across the studies, potentially influencing the reported RFS and OS rates. And among the included studies, the follow-up time of ETV group was longer than TDF group. Due to the insufficient follow-up time in the TDF group, it is difficult to compare the recurrence rate of HCC. Further large sample, prospective, and multicenter studies are needed to clarify the clinical benefits of recently approved antiviral drugs for HBV-related HCC patients. Despite these limitations, the findings have important clinical implications. Clinically, this supports the continued use of these drugs as first-line treatments for chronic HBV infection in HCC patients. However, further studies are needed to explore which treatment might be more effective in specific subgroups, such as patients with advanced liver cirrhosis or those undergoing liver transplantation.

In conclusion, this meta-analysis suggested that TDF was associated with reduced risk of late recurrence and improved survival in patients with HBV-related HCC after surgical resection compared with ETV. Therefore, based on current evidence, TDF could be preferentially recommended after surgical resection for patients without contraindications. However, further large-scale and prospective studies are needed to validate the current result, and investigate the role of TAF in the postoperative prognosis of HBV-related HCC.

## Data Availability

The original contributions presented in the study are included in the article/**Supplementary Material**. Further inquiries can be directed to the corresponding author.
