# Supplementary material for: Tenofovir disoproxil fumarate versus entecavir on the prognosis of hepatitis B virus-related hepatocellular carcinoma after surgical resection: a systematic review and meta-analysis
Source: Front Oncol. 2025 May 27;15:1462794. doi: 10.3389/fonc.2025.1462794 (PMC12149102; doi:10.3389/fonc.2025.1462794)
Supplement: Supplementary file 1 [file Table1.docx]

Supplementary Table 1. Cochrane review for risk of bias

| study | Random sequence generation (selection bias) | Allocation concealment (selection bias) | Blinding of participants and personnel (performance bias) | Blinding of outcome assessment (detection bias) | Incomplete outcome data (attrition bias) | Selective reporting (reporting bias) | Other bias |
| --- | --- | --- | --- | --- | --- | --- | --- |
| He et al, 2023 | Low | Low | Low | Low | Low | Low | Low |

Supplementary Table 2. NOS criteria for cohort study

| study | Representativeness of the exposed cohort | Selection of the non-exposed cohort | Ascertainment of exposure | Demonstration that the outcome of interest was not present at start of study | Comparability of cohorts on the basis of the design or analysis | Assessment of outcome | Was follow-up long enough for outcome to occur | Adequacy of follow-up of cohorts | Total quality score |
| --- | --- | --- | --- | --- | --- | --- | --- | --- | --- |
| Qi et al, 2021 | ☆ | ☆ | \ | \ | ☆ | ☆ | ☆ | \ | 5 |
| Shen et al, 2021 | ☆ | ☆ | ☆ | ☆ | ☆ | ☆ | ☆ | \ | 7 |
| Wu et al, 2021 | ☆ | ☆ | ☆ | \ | ☆ | ☆ | ☆ | \ | 6 |
| Zhang et al, 2018 | ☆ | ☆ | ☆ | ☆ | ☆ | ☆ | ☆ | \ | 7 |
| Tsai et al, 2021 | ☆ | ☆ | ☆ | ☆ | ☆☆ | ☆ | ☆ | \ | 8 |
| Wang et al, 2022 | ☆ | ☆ | ☆ | ☆ | ☆☆ | ☆ | \ | \ | 7 |
| Choi et al, 2021 | ☆ | ☆ | ☆ | ☆ | ☆☆ | ☆ | \ | ☆ | 8 |
| Lee et al, 2021 | ☆ | ☆ | ☆ | ☆ | ☆☆ | ☆ | ☆ | ☆ | 9 |
| Yun et al, 2022 | ☆ | ☆ | ☆ | ☆ | ☆ | ☆ | ☆ | \ | 7 |
| Li et al, 2023 | ☆ | ☆ | ☆ | ☆ | ☆☆ | ☆ | ☆ | \ | 8 |
| Kao et al, 2023 | ☆ | ☆ | ☆ | ☆ | ☆ | ☆ | ☆ | \ | 7 |
| Chang et al, 2024 | ☆ | ☆ | ☆ | ☆ | ☆ | ☆ | ☆ | \ | 7 |
| Chung et al, 202**5** | ☆ | ☆ | ☆ | ☆ | ☆☆ | ☆ | ☆ | ☆ | 9 |
| Kong et al, 2024 | ☆ | ☆ | ☆ | ☆ | ☆ | ☆ | ☆ | ☆ | 8 |
